# The COVID‐19 infection control response at a large stand‐alone comprehensive cancer center in Los Angeles County

**DOI:** 10.1002/cnr2.1669

**Published:** 2022-07-01

**Authors:** Hannah Battey, Brenna Doran, Annemarie Flood, Juliet Nussbaum, Tyler Seto, Suwannee Srisatidnarakul, Bernard Tegtmeier, Sanjeet Dadwal

**Affiliations:** ^1^ Department of Infection Prevention and Control City of Hope National Medical Center Duarte California USA; ^2^ Department of Quality, Risk and Regulatory Management City of Hope National Medical Center Duarte California USA; ^3^ Division of Infectious Diseases, Department of Medicine City of Hope National Medical Center Duarte California USA

**Keywords:** cancer prevention, epidemiology and prevention, hematological cancer, medical oncology

## Abstract

**Background:**

The City of Hope National Medical Center (COH) is the only stand‐alone comprehensive cancer center in Los Angeles, a county that was deemed a COVID‐19 pandemic epicenter at the height of the 2020 winter surge. The immunocompromised patient population frequently experienced delays in infection control guidelines from local and government bodies due to minimal data available in comparison to the general population. This required COH to make swift, informed decisions for the best interest of the patient population.

**Aim:**

Here, we review the comprehensive COVID‐19 infection control response conducted at COH within the context of a high‐risk patient population, predominately comprised of patients with hematologic malignancies.

**Methods and Results:**

This infection control response focused on prevention of COVID‐19 transmission on campus, COVID‐19 testing, and isolation management. These efforts consisted of COVID‐19 screening, limitation of personnel on campus, source control, contact tracing, COVID‐19 vaccination, establishment of in‐house testing and implementation and management of COVID‐19 testing. Between January 2020 and September 2021, COH implemented a robust in‐house testing program, completed well over 1000 contact traces, ensured COVID‐19 vaccinations were distributed to all eligible staff and patients, and established an algorithm for COVID‐19 infection resolution, all without compromising the number of hematopoietic stem cell transplants (HCTs) performed, surgical volume, or healthcare‐associated standardized infection ratios (SIR).

**Conclusion:**

Institutional collaboration and attention to infection control was pivotal to minimizing the burden of the COVID‐19 pandemic.

## 1 INTRODUCTION

On January 5, 2020, the World Health Organization (WHO) published notification of the COVID‐19 outbreak caused by Severe Acute Respiratory Syndrome—Coronavirus 2 (SARS—CoV‐2). On March 11, 2020, the WHO declared COVID‐19 a global pandemic and by the end of 2020, California experienced the highest number of reported cases of COVID‐19 in the United States, with LA County leading the state with the highest number of deaths per capita.^1^ On January 1, 2021, the population of 10 million individuals reached more than 820 000 confirmed COVID‐19 cases, with a testing positivity rate over 20%.^2^

The City of Hope National Medical Center (COH) is one of 51 comprehensive cancer centers in the United States and the only stand‐alone cancer institution located in LAC. Founded in 1913, the 217‐bed academic and research‐oriented medical center performs more than 700 hematopoietic stem cell transplants (HCTs) and immune effector cell (IEC) administrations annually, with an active hematologic malignancy (HM) and solid tumor (ST) program (inpatient days: 66% HM and 33% ST, respectively) with over 10 000 surgical and endoscopic procedures performed each year.

COVID‐19 disease presentation is highly varied and that holds true for patients with cancer.^3^, ^4^ Immunocompromised patients with COVID‐19 are at a higher risk for severe COVID‐19 disease.^3^, ^5^ The COVID‐19 recommendations initially released by the Centers for Disease Control and Prevention (CDC) and LACDPH were primarily described for the general population, leaving little applicable guidance for the immunocompromised cancer patient population. COH created a COVID‐19 task force comprised of institutional leaders, who implemented and managed the evolving COVID‐19 response. Within this task force, a Command Center of individuals from infection prevention (IP) and infectious disease (ID) conducted daily huddles to discuss challenges, disseminate information, and implement action plans. This team consulted with all stakeholders and established policies and practices, with a focus on minimizing delays in critical cancer care and maximizing patient and employee safety. In this paper, we discuss the expeditious decision making and pandemic planning demonstrated by the COH Duarte main campus COVID‐19 task force that was instrumental to the creation, management, and implementation of the pandemic infection control response.

## 2 METHODS

The COVID‐19 infection control response described in this paper took place beginning in January 2020, when the World Health Organization (WHO) announced the SARS‐CoV‐2 outbreak, up through September 2021. This response was focused on prevention of COVID‐19 transmission on campus, COVID‐19 testing, and isolation management.

### 2.1 Prevention of COVID‐19 transmission on campus

#### 2.1.1 COVID‐19 screening, limitation of personnel on campus and universal masking

In early January 2020, COH began dissemination of the WHO SARS‐CoV‐2 public health notice throughout the hospital. All admitting personnel and outpatient clinic frontline staff were provided with patient screening scripts that assessed for recent travel to Wuhan, China and onset of fever or new upper respiratory symptoms. In response to the global spread of the virus, the travel screening question was later expanded to include any recent international travel.

Initially, all patients that screened positive for recent travel or new onset of respiratory symptoms were assessed by ID and IP and those who met “person under investigation (PUI)” criteria underwent a nasopharyngeal swab and RT‐PCR for detection of SARS‐CoV‐2. Due to the frequent positive screening and limited infectious disease staff available, the COVID‐19 Command Center constructed and disseminated a series of COVID‐19 algorithms that standardized the process of COVID‐19 testing based on the patient location and service line (pediatrics/adult). We also developed a dedicated space (Fever Respiratory Clinic—FRC) to evaluate patients considered as PUI to ensure standardized evaluation and limit exposure in the clinics, which was staffed by advanced practice providers (APPs) with ID support.

On March 15, 2020, COH officially implemented an expansive work from home program, days before the statewide stay at home order was announced. On March 17, COH began daily symptom/temperature checks and exposure screening of all employees, contractors, vendors, volunteers, and patients to prevent the entry of symptomatic individuals into the hospital. On March 25, vendor and volunteer activities were placed on hold and inpatient visitation was restricted. Universal masking began on March 27 as a critical means of source control, again preceding public health mandates.

#### 2.1.2 Contact tracing

Contact tracing of COVID‐19‐positive individuals was recommended by the CDC at the onset of the pandemic as a means of minimizing the spread of the virus. The organization began contact tracing with its first PUI in February 2020. The contact tracing process was conducted in accordance with CDC guidelines, wherein all COH employees and patients identified as close contacts of confirmed COVID‐19‐positive individual were notified. Those with a medium‐ or high‐risk exposure were quarantined until clearance (via test‐based or time‐based strategy). Medium‐ and high‐risk exposures were characterized per CDC guidelines and included an assessment of the PPE worn at the time of exposure, and whether the COVID‐19‐positive patient had an aerosol generating procedure performed.^6^

In February 2020, the CDC defined a close contact as any individual within 6 feet of an infected person for 2 or more minutes, with the infectious window beginning 2 days prior to symptom onset. In June 2020, COH instituted mandatory universal eye protection in clinical spaces, ahead of the recommendation from LAC and CDC. In October 2020, the CDC's close contact definition was further expanded to a cumulative 15‐min time interval over a 24‐h period. CDC also shortened the quarantine guidance from 14 to 10 days in December 2020. Following the adoption of asymptomatic employee testing, employees were offered a voluntary COVID‐19 test at day 7 of quarantine, which allowed them to return to work early with a negative result to reduce the impact of staff shortages. On September 1, 2021 California Department of Public Health (CDPH) returned to a more conservative return to work recommendation of 10 days from the date of exposure, due to an increase in the number of cases of COVID‐19 and the circulating delta variant, and COH followed suit.

#### 2.1.3 COVID‐19 vaccination

COH began administering COVID‐19 vaccinations to HCW on December 16, 2020. COH implemented a mandatory COVID‐19 vaccination program that coincided with the CDPH COVID‐19 vaccine requirement for all HCW. As of the September 30, 2021, CDPH deadline for vaccination of all HCW, the institution's robust vaccination efforts resulted in 87% fully vaccinated staff (excluding terminated COH employees and contractors), with the remaining staff documented as partially vaccinated or unvaccinated. All individuals with an approved exemption (medical, religious) were required to test twice weekly for COVID‐19 and provided a N95 mask to wear at work. COH constructed a comprehensive contingency plan in anticipation of the expected loss of workforce from the newly instituted vaccine requirement.

Many considerations were made for vaccinating COH patients early on, since vaccines were a scarce resource and the immunocompromised status of patients would determine the potential effectiveness of the COVID‐19 vaccine. Prior to the March 15, 2021, LAC guidance that recommended vaccination of cancer patients >16 years of age, the COH COVID‐19 Task Force constructed a tiered patient prioritization system. Utilizing the Los Angeles County vaccine hierarchy^7^ as a template, COH aimed to vaccinate patients that were at highest risk of developing severe disease if exposed.

### 2.2 COVID‐19 testing

#### 2.2.1 Establishing and operationalizing in‐house testing

The in‐house DiaSorin Simplexa® COVID‐19 molecular testing method was established on March 31, 2020. One of the greatest logistical challenges resulting from the increased frequency of COVID‐19 testing was the requirement of negative pressure patient rooms. COH has 132‐positive pressure patient rooms, 12 negative pressure patient rooms and 71 net neutral patient rooms. A multidisciplinary team that included nursing, IP, Quality, Facilities and Support Services was assembled in April 2020 to plan and construct the necessary infrastructure for the anticipated surge of COVID‐19 patients. A unit of neutral pressure rooms in one of the hospital wings was transformed into the COVID‐19 Response Unit (CRU). Negative pressure rooms were created by removing the windowpanes and installing a plexiglass fitted HEPA filtration system in each room, with monometers to continually monitor the negative pressure differential. Temporary barriers were built to physically delineate COVID‐19 and non‐COVID‐19 patient care areas. The number of rooms within the CRU fluctuated, ranging from 1 to 25 rooms. Following the end of the winter surge, the CRU was disbanded on June 1, 2021 due to sustained low COVID‐19 patient volume. Going forward, patients with COVID‐19 were housed in negative pressure rooms in the hospital.

#### 2.2.2 The evolution and implementation of testing criteria

The COVID‐19 testing program was composed of pre‐admission, day 6, pre‐HCT, PUI and pre‐procedure (outpatient) testing. The goal of this testing strategy was to (1) prevent delays in cancer treatment, (2) prevent delays in the triage and management of symptomatic patients with COVID‐19, (3) limit nonessential appointments for asymptomatic/mildly symptomatic COVID‐19‐positive individuals, and (4) continue treatment as usual for COVID‐19‐negative patients. All patients were tested for COVID‐19 within 72 h prior to admission and on day 6 of admission, with the date of admission as day 1. The day 6 testing was intended to identify any patients who might have been exposed to COVID‐19 immediately prior to admission and were in the incubation period during the initial admission testing event. Patients were also tested the day prior to lymphodepletion/conditioning and the day prior to transplant/immune effector cell administration. Any patient admitted with symptoms of new‐onset fever, cough, or shortness of breath in the prior 14 days was treated as a PUI and placed on enhanced droplet/contact isolation until they met clearance criteria. Asymptomatic individuals were tested prior to selected high‐risk procedures (≤3 days), following a COVID‐19 exposure, or if required for clinical trials.

Beginning August 2, 2020, COH began offering voluntary COVID‐19 testing to employees working in high‐risk areas (e.g., CRU, FRC, Evaluation Treatment Center, operating room) and symptomatic employees. On November 24, 2020, COH began offering voluntary weekly testing for asymptomatic staff, which preceded the December 14 CDPH mandate requiring acute care hospitals to provide access to weekly HCW testing.

### 2.3 COVID‐19 isolation management

COH primarily provides services to cancer patients with hematological and/or solid tumor malignancies at varying stages of illness and immunocompromised status. This spectrum of immunocompromised status made establishment of a clear COVID‐19 infection window challenging. To address this, COH implemented a COVID‐19 infection banner system in the electronic medical record to identify infectious patients and constructed an algorithm to manage banner resolution. The COH infection banner algorithm consisted of an assessment of the patient's medical history and the COVID‐19 infectious period was categorized into a 14/20 or 14/28/7‐day interval testing frequency, with the start date of symptoms established as day 1 (Table 1). The 14/20‐day testing strategy was implemented for solid organ cancer patients on active chemotherapy/immunotherapy within the last 4 weeks or about to start chemotherapy, and all other outpatients that did not meet 14/28/7‐day criteria. The 14/20‐day testing involved waiting 14 days from start of symptoms and testing, with a negative test resulting in infection banner resolution and a positive test allowing for infection banner removal without a test at day 21. The 14/28/7‐day testing model was utilized for all IEC and HCT patients T‐25 to T + 100 (where T = day of transplant/IEC), patients with acute or chronic graft versus host disease (GVHD), hematologic malignancy patients within the last 4 weeks or about to start chemotherapy/immunotherapy. The 14/28/7‐day testing allowed for potential infection banner resolution on day 14 if a negative result was received, with a positive test requiring follow‐up on day 28 for consideration of banner removal. If the day 28 test was also positive, testing was performed in 7‐day increments until a negative test was achieved. Two negative tests were required for infection discontinuation.

**TABLE 1:** The COH algorithm for removing the COVID‐19 isolation banner from the electronic medical record for hematological malignancies/HCT and solid organ cancer patients

Scenario	14/20^a^	14/28/7^a^ ^,^ ^b^
IEC and transplant patients *T*‐25 to *T* + 100 (where *T* = day of transplant or IEC)		X
Active acute or chronic GVHD, regardless of time after HCT		X
Hematologic malignancy patients that are about to receive or have received chemotherapy/immunotherapy within the last 4 weeks		X
Solid organ cancer patients with chemotherapy/immunotherapy within last 4 weeks	X	
Solid organ cancer patients about to start chemotherapy/immunotherapy	X	
All other outpatients	X	

*Notes*: The 14/20‐day testing strategy was utilized for solid organ cancer patients and all other non‐hematological malignancy patients: wait 14 days from start of symptoms and test; if test negative then proceed, if positive then return without test at day 21. The 14/28/7‐day testing strategy was utilized for IEC, transplant, acute/chronic GVHD, and hematologic malignancy patients: wait 14 days from start of symptoms and test; if negative then proceed, if positive then retest at day 28. If negative then proceed, if positive then retest every 7 days until negative. If the patient had no symptoms, then the date of positive test was used as day 1. For inpatients qualifying for the 14/28/7‐day testing strategy, 2 negative tests were required to discontinue isolation. ID and IP monitored and removed COVID infection banners when patients met criteria.

^a^ If patient has no symptoms, then use the date of the test.

^b^ Inpatients in the CRU need 2 negative tests 24 h apart prior to transfer to another inpatient unit. A negative test is not required for discharge to home.

## 3 RESULTS

**FIGURE 1:**
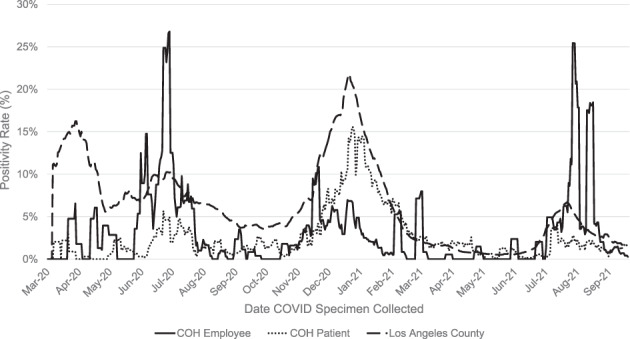
COVID‐19 positivity rate for patients and employees tested at City of Hope in comparison to the Los Angeles County positivity rate between March 2020 and September 2021. The positivity rate was calculated as the percentage of all COVID‐19 tests that were positive in 7‐day average increments, using the date of specimen collection. LA County data includes reported cases from healthcare institutions and laboratories within the defined county lines. This data is publicly available^2^

**FIGURE 2:**
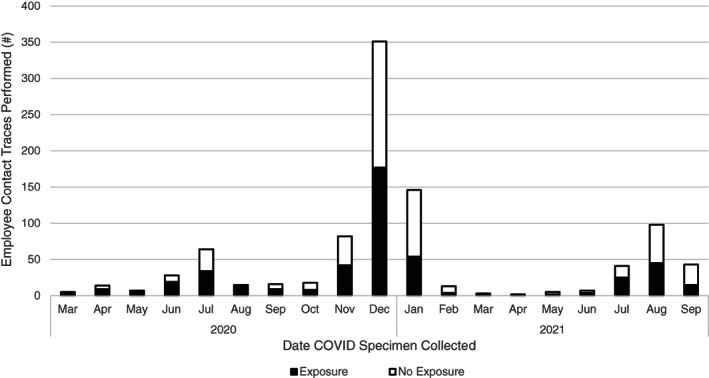
The number of employee contact traces completed between March 2020 and September 2021, classified as exposure versus no exposure. An exposure was defined based on CDC guidelines (proximity/time‐based assessment of interaction with the COVID‐19 index case). All contact traces were performed by the COH IP Department

The peak positivity rate (7‐day rolling average) for patients and employees occurred in July 2020 (patients: 5.7%, employees: 26.8%) and December 2020/January 2021 (patients: 15.3%, employees: 6.8%), mirroring the trend seen in LAC (July: 10%, December/January: 21%) (Figure 1). The IP department completed nearly 600 employee exposure traces and over 200 patient exposure traces in 2020. Between November 2020–January 2021 alone, 579 employee traces were completed, of which 273 were classified as an exposure that required employee/patient notification (Figure 2). Of note, approximately half of all cases documented during this time were nonexposure cases.

**FIGURE 3:**
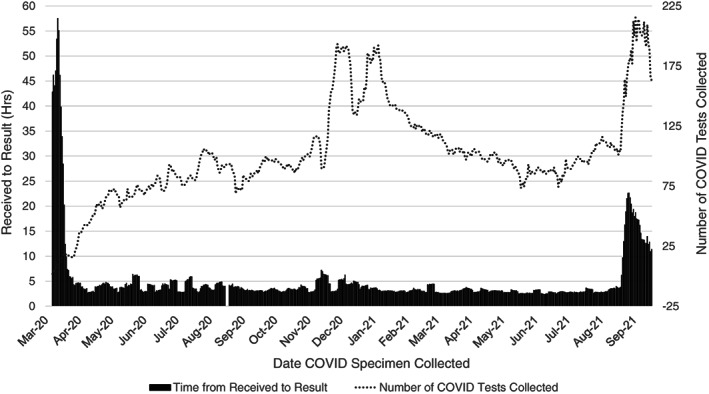
Average time in hours from laboratory receival to result for patient and employee COVID‐19 testing compared alongside the number of daily COVID‐19 tests collected. The time to result and COVID‐19 testing numbers were averaged in 7‐day increments. These data include both internal and external laboratory tests for Duarte main campus

The introduction of the in‐house DiaSorin Simplexa® COVID‐19 molecular testing method on March 31, 2020 drastically reduced the turnaround time (TAT) of laboratory results and expanded the total number of COVID‐19 tests collected (24‐h maximum: *n* = 302 – January 12, 2021) (Figure 3). Prior to the adoption of this testing modality, COVID‐19 specimens were sent to external labs (e.g., reference laboratories, public health laboratories and CDC), with TAT in excess of 83 h. The in‐house testing method resulted in consistent same‐day TAT between 4 and 5 h. On August 24, 2021, the CDPH mandated twice weekly COVID‐19 testing for unvaccinated HCW, which noticeably increased the total number of tests collected (24‐h maximum: n = 332 – September 22, 2021) and TAT (28‐h average TAT − September 9, 2021). However, these labs were sent to a reference laboratory, with no negative impact on the in‐house testing program for patients or symptomatic staff.

**FIGURE 4:**
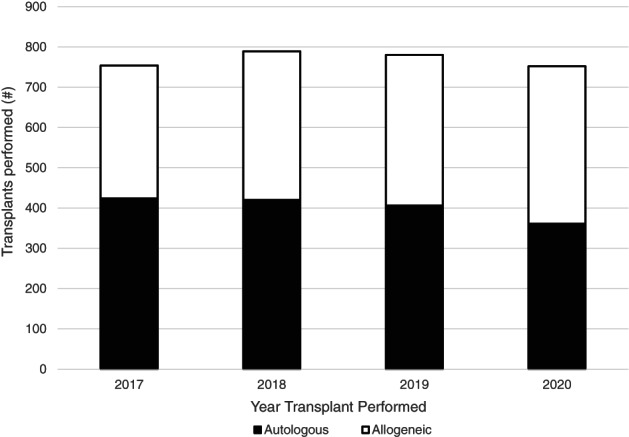
Number of hematopoietic stem cell transplants (HCTs) performed at COH between 2017 and 2020, broken down by autologous and allogeneic

**FIGURE 5:**
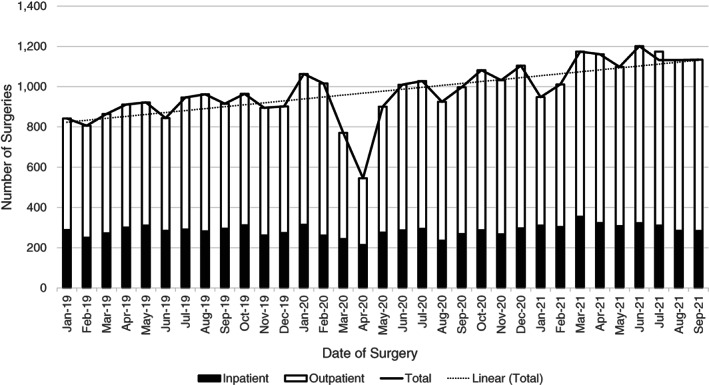
The number of inpatient and outpatient surgical and endoscopic procedures performed on COH Duarte campus between January 2019 and September 2021. The linear trendline associated with the total number of inpatient and outpatient surgeries illustrates a steady increase in surgeries over time

Despite the COVID‐19 pandemic, COH continued to maintain stable patient volumes and perform essential HCTs (Figure 4). COH performed 752 autologous and allogeneic procedures in 2020, a minimal decrease compared to the 780 procedures performed the year prior. Despite the onset of the COVID‐19 pandemic in 2020, COH exceeded the number of surgeries performed in 2019 (2019: *n* = 10 773; 2020: *n* = 11 474) (Figure 5). Despite a noticeable dip in (primarily outpatient) surgeries in April 2020, COH quickly recovered its surgical volume and superseded the number of cases performed in 2019. COH stepped up to the challenge of providing essential, life‐saving cancer interventions to its high‐risk patient population throughout the entirety of the COVID‐19 pandemic.

## 4 DISCUSSION

Healthcare infection control responses have been crucial for mitigating the risk of transmission in healthcare facilities of all sizes and patient populations served. Due to the limited information available for immunocompromised patients regarding infectivity and transmissibility after symptom resolution, we were required to make swift decisions to protect the safety of our patients and healthcare teams. This involved minimizing potential exposure to COVID‐19‐positive individuals by limiting the number of people on campus, screening all patients and employees, testing in designated areas (FRC and drive through), conducting contact tracing to ensure all individuals exposed to COVID‐19 were quarantined appropriately and ensuring that staff were provided with adequate PPE supplies. The limitation of inpatient visitation altered the means in which families were able to support their loved ones, a particularly challenging reality for patients battling cancer.

The CDC's close contact definition changed several times throughout the pandemic, which significantly mitigated the contact tracing burden over time. COH's implementation of universal eye protection and widespread staff compliance with PPE further decreased the number of medium/high‐risk exposures. Offering symptomatic and asymptomatic testing to employees and maintaining contact tracing throughout the entirety of the pandemic was crucial for minimizing exposures on campus. COH placed a large focus on the COVID‐19 vaccination program, considering that vaccination of healthcare workers (HCW) and family members of cancer patients is a critical component of preventing infection transmission in this population, since these patients may not be capable of mounting an adequate response to vaccination themselves.

Early in the COVID‐19 pandemic, it became increasingly apparent that utilizing a rapid‐in‐house COVID‐19 testing methodology was essential for minimizing delays in patient care, timely discontinuation from isolation and preserving PPE supplies. The implementation of the COVID‐19 inpatient and outpatient testing protocol increased the number of COVID‐19 tests performed over time without compromising the TAT. Additionally, the construction of the FRC and CRU expanded the capacity for COVID‐19 patients, which became critical during the surge periods.

Initially, the CDC defined the COVID‐19 infectious period as the 14 days following symptom onset or date of positive test, whichever came first. While SARS‐CoV‐2 can be detected by PCR for several months following disease onset,^8^, ^9^, ^10^ detection of viral RNA does not imply that the virus is viable and infectious, only that viral RNA is present. Several months into the pandemic, the CDC shortened the infectious period for persons with mild to moderate symptoms to 10 days. However, it is known that patients with HCT may shed respiratory viruses (RSV, rhinovirus, endemic coronaviruses) for prolonged periods of time.^11^ It was not until February 2021 that the CDC extended the infectious period for persons with severe illness or with immunocompromised status to 20 days. Prior to these recommendations, COH established the COVID‐19 isolation management algorithm for differentiating isolation resolution between solid tumor and hematological malignancy patients and separating patients with short versus prolonged viral shedding. However, the algorithm had its limitations given the numerous caveats of disease status in immunocompromised patients. Patients with hematological malignancies demonstrated the ability to present with protean symptoms and shed viral RNA intermittently for extended periods of time, reverting between positive and negative status repeatedly as patients underwent multiple pre‐procedure and pre‐admission testing episodes. Additionally, several patients presented as nasopharyngeal negative and bronchoalveolar lavage positive; a previously documented phenomena of immunocompromised patients.^12^, ^13^ This may also be a result of higher viral loads in lower respiratory specimens.^12^, ^14^ Since many COVID‐19 symptoms overlap with cancer‐related symptoms, relying on the symptom‐based strategy alone was not feasible. PCR cycling times were one of the many factors taken into consideration to determine the infectiousness and corresponding treatment eligibility. For COVID‐19 patients with deviations from the typical disease presentation, careful consideration and coordination between ID physicians, the care team, and IP occurred to ensure isolation status monitoring was conservatively upheld when necessary.

Although COH's IP team was charged with several additional COVID‐related daily tasks, the department's surveillance efforts and healthcare‐associated infection (HAI) reduction interventions were not compromised by the pandemic. The standardized infection ratio (SIR) compares the actual number of HAIs at each hospital, to the predicted number of infections, while adjusting for various facility and/or patient‐level factors that contribute to HAI risk within each facility. Amidst the COVID‐19 pandemic, COH improved performance in prevention of catheter‐associated urinary tract infections (SIR: 2019 = 0.799, 2020 = 0.364) and hospital‐acquired *Clostridium difficile* infections (SIR: 2019 = 1.331, 2020 = 1.125). The SIR for central‐line associated bloodstream infections was increased in 2020 (SIR: 1.020) compared to 2019 (SIR: 0.867), but still not above what would be expected in this high‐risk patient population. The institution experienced a 10% increase in overall inpatient hand hygiene compliance in 2020 (85%) compared to 2019 (75%), which may partially explain the improved SIRs. Additionally, the proactive and comprehensive infection control response at COH was paramount to preventing the spread of COVID‐19, with no documented nosocomial patient to patient or staff to patient transmission as of September 2021. This is impressive considering that nosocomial transmission of COVID^15^, ^16^, ^17^ and other common respiratory viruses^18^, ^19^ has been documented.

Every department was affected by the pandemic, but despite the challenges, rose to contribute to the team. Institutional collaboration was pivotal to minimizing the burden of the COVID‐19 pandemic at COH.

## AUTHOR CONTRIBUTIONS

**Brenna Doran:** Methodology (supporting); writing – review and editing (supporting). **Annemarie Flood:** Methodology (supporting); writing – review and editing (supporting). **Juliet Nussbaum:** Methodology (supporting); writing – review and editing (supporting). **Tyler Seto:** Methodology (supporting); writing – review and editing (supporting). **Suwannee Srisatidnarakul:** Methodology (supporting); writing – review and editing (supporting). **Bernard Tegtmeier:** Data curation (supporting); methodology (supporting); validation (supporting); writing – review and editing (supporting). **Sanjeet Dadwal:** Conceptualization (equal); project administration (equal); supervision (equal); writing – review and editing (equal).

## CONFLICT OF INTEREST

All authors declare no conflict of interest.

## ETHICS STATEMENT

This is an IRB exempt study.

## Data Availability

The data that support the findings of this study are available from the corresponding author upon reasonable request.
